# Feeder Cell Detachment in Drug Response Profiling of Leukemia Cell Coculture Can Be Prevented by Conditioned Medium

**DOI:** 10.1002/cam4.71070

**Published:** 2025-07-19

**Authors:** Ivana Vonkova, Olga Martinkova, Jitka Stancikova, Ctibor Skuta, Ondrej Hrusak, Petr Bartunek

**Affiliations:** ^1^ CZ‐OPENSCREEN, National Infrastructure for Chemical Biology, Institute of Molecular Genetics of the Czech Academy of Sciences Prague Czech Republic; ^2^ CLIP, Second Faculty of Medicine Charles University Prague Czech Republic; ^3^ Department of Pediatric Hematology and Oncology University Hospital Motol Prague Czech Republic; ^4^ Laboratory of Cell Differentiation Institute of Molecular Genetics of the Czech Academy of Sciences Prague Czech Republic

**Keywords:** conditioned medium, drug response profiling, mesenchymal stromal cells

## Abstract

**Background:**

Determining drug sensitivity in tumor cells ex vivo is a frequently used method not only in leukemia cell research but also serves as an indispensable tool for searching for alternative treatment strategies for leukemia patients unresponsive to classical treatment. The drug response profiling (DRP) method based on fluorescence imaging of leukemia cells cocultured with a monolayer of mesenchymal stromal cells (MSC) and treated with antileukemia drugs currently represents one of the most powerful methods well suited for simultaneous screening of multiple drugs with significantly reduced numbers of cells and time required to perform the test. Moreover, the coculture of leukemia cells with MSC simulates the situation in the bone marrow and thus improves the prediction accuracy of drug sensitivity in vivo. However, the addition of leukemia cells frequently leads to MSC detachment from the well bottom, which disturbs automated image analysis and, in the end, may lead to imprecise DRP results.

**Results:**

The MSC detachment, either mild or strong, was observed in 27.8% of our experiments. To overcome this phenomenon, we introduced a simple solution for effectively preventing the MSC detachment. It is based on the addition of MSC‐conditioned medium to leukemia cells‐MSC coculture. This method improvement reduced the proportion of experiments impaired by the strong MSC detachment from 16.7% to 3.3%. Importantly, implementing MSC‐conditioned medium had no adverse effect on the leukemia cell viability or drug response.

**Conclusions:**

Upgrading the DRP method with the simple step of addition of MSC‐conditioned medium to the leukemia cells–MSC coculture significantly reduces cases of MSC detachment without interfering with any advantages of this DRP method, including its feasibility for high‐throughput screening.

**Abbreviations**
ALAL: acute leukemias of ambiguous lineage
AML: acute myeloid leukemia
B‐ALL: B lymphoblastic acute leukemia
CM: conditioned medium
CQD: CyQUANT Direct Cell Proliferation Assay
DRP: drug response profiling
MSC: mesenchymal stromal cells
T‐ALL: T lymphoblastic acute leukemia
UM: unconditioned medium

## 1 Introduction

Ex vivo drug response profiling (DRP) is a powerful tool for uncovering the sensitivity to chemotherapeutics in tumor cells. It is used for leukemia cell research and to select rescue therapy in patients who fail to respond to classical treatment [1]. The focus of this study is on high‐throughput DRP of pediatric acute leukemias: T lymphoblastic acute leukemia (T‐ALL), B lymphoblastic acute leukemia (B‐ALL), acute myeloid leukemia (AML), and acute leukemias of ambiguous lineage (ALAL). Drug resistance can be investigated by several assays, including flow cytometry‐based and 3‐(4, 5‐dimethylthiazolyl‐2)‐2, 5‐diphenyltetrazolium bromide (MTT) assays [2, 3, 4]. However, these assays have limited throughput due to complicated procedures and high requirements for cell material. We adapted the protocol established by Frismantas et al. [5], which is based on automated microscopic imaging and is well suited for high‐throughput DRP. The method involves coculturing the leukemia cells with the bone marrow mesenchymal stromal cells (MSC) in a medium with tested chemotherapeutics. MSC are essential for keeping leukemia cells in optimal condition throughout the entire DRP process. Although the DRP assay is straightforward, some steps could be further optimized. Here, we describe a phenomenon of MSC detachment from the well bottom upon the addition of leukemia cells, its consequences for the assay output, and measures that can be applied to reduce its frequency.

## 2 Materials and Methods

The aim of this study was to improve an existing DRP method in order to overcome frequent problems with unwanted MSC detachment triggered by addition of leukemia cells to the coculture.

### 2.1 Patient Samples

In this study, we used patient samples of all main childhood acute leukemia types (T‐ALL, B‐ALL, and AML) as well as samples of ALAL. Patient samples were obtained with the informed consent of the patient's guardians. The study was approved by the Ethical Committee of the Second Faculty of Medicine, Charles University in Prague (ref. no. EK‐602.32/22; agreement emitted on May 15, 2022) and conducted in accordance with the Declaration of Helsinki. An overview of samples used in experiments presented in this study could be found in Table S1.

### 2.2 DRP Assay Design and Setting

The general pipeline for the DRP assay was adapted from the method described in the publication of Frismantas et al. [5] and it is summarized in Figure S1. The DRP assay was performed on 1536‐well plates (Corning, 3838, NY, USA). The source of leukemia cells was a liquid nitrogen‐stored density gradient enriched bone marrow or peripheral blood diagnostic sample containing a minimum of 80% leukemia cells. As a supporting feeder layer, an immortalized human bone marrow mesenchymal cell line—hTERT cell line (MSC; Cat. No. T0523, Applied Biological Materials Inc., Richmond, Canada) was used.

DRP was assessed in MSC‐leukemia cell cocultures in serum‐free screening medium (CTS AIM‐V, Thermo Fisher Scientific, MA, USA). Prior to the setup of the DRP experiments, MSC were grown in culture medium (Advanced RPMI 1640, Gibco, Thermo Fisher Scientific, MA, USA; supplemented with 10% inactivated FBS, 1% Penicillin–Streptomycin, and 1% GlutaMAX [both Gibco, Thermo Fisher Scientific, MA, USA]) on 10 cm tissue culture dishes (TPP, Trasadingen, Switzerland). After reaching the cell number required for the DRP assay, MSC were harvested using Trypsin–EDTA (Gibco, Thermo Fisher Scientific, MA, USA), washed with PBS, and resuspended in screening medium to a density 225 cells/μL. MSC (900 cells per well) were then plated in 4 μL of screening medium per well and left for 24 h to attach. Subsequently, leukemia cells (5000 cells per well) were added in 2 μL of screening medium to the wells with attached MSC, and the coculture was left for another 24 h. Chemical compounds dissolved in dimethyl sulfoxide (DMSO) were then added in several concentrations (in the range from 3 × 10^−14^ M to 1 × 10^−4^ M, each concentration point in duplicates or triplicates) into individual wells of the 1536‐well plate using acoustic liquid transfer (Echo, Beckman Coulter, CA, USA) and the cells were incubated with the compounds for 72 h. Despite the MSC–leukemia cells being cocultured for a long period of time (in total 96 h), no special treatment of MSC to reduce their proliferation and consequently to prevent feeder layer overgrowth was required. The serum‐free screening medium significantly reduced the proliferation of MSC, a phenomenon previously described by others [6]. The reduced proliferation of MSC did not prevent their ability to fulfill their function as a support for leukemia cells during the DRP experiment. The average survival rate of untreated leukemia cells at the end of the DRP experiment was 52.4% in coculture with MSC and only 3% without MSC. After 72 h of incubation with chemical compounds, living cells were stained by CyQUANT Direct Cell Proliferation Assay (Thermo Fisher Scientific, MA, USA) and automatically imaged (Operetta, PerkinElmer/Revvity, MA, USA). Acquired images were analyzed using Columbus software (PerkinElmer/Revvity, MA, USA). For each compound, a response curve was calculated using relative inhibition (proportion of maximal efficacy) as a readout and the LC50, concentration when half of the maximal efficacy was reached (relative inhibition = 0.5), served as the main parameter for describing drug potency.

### 2.3 Production and Use of Conditioned Medium

MSC at a density of 0.5 × 10^6^ cells/mL were seeded in culture medium on 10 cm dishes 6 days prior to the start of the DRP experiment. When 70% confluency was reached (about 3 days after seeding), culture medium was discarded and replaced with serum‐free screening medium after a brief rinse of the MSC with PBS. After 3 days, the medium was collected, centrifuged to remove any detached MSC, and directly used to dilute freshly defrosted leukemia cells. The leukemia cell suspension was then added to the pre‐attached MSC culture on the 1536‐well plate, resulting in a mixture of fresh and conditioned screening medium (volume ratio 2:1), a setup referred to as conditioned medium in this paper. The setting referred to as unconditioned medium used fresh medium.

### 2.4 Statistics

Statistical significance of differences between cell counts was assessed using unpaired *t*‐test and *F*‐test to compare variances. The significance level of *p* < 0.05 indicates a statistically significant difference, * denotes *p* < 0.05, ** denotes *p* < 0.01, *** denotes *p* < 0.001, and **** denotes *p* < 0.0001, while ns signifies no statistical difference. Correlations were assessed using Pearson's correlation coefficient (R squared). All statistical calculations were performed in GraphPad Prism version 10.2.2.

## 3 Results

### 3.1 MSC Detachment in a Coculture of MSC With Leukemia Cells

**FIGURE 1:**
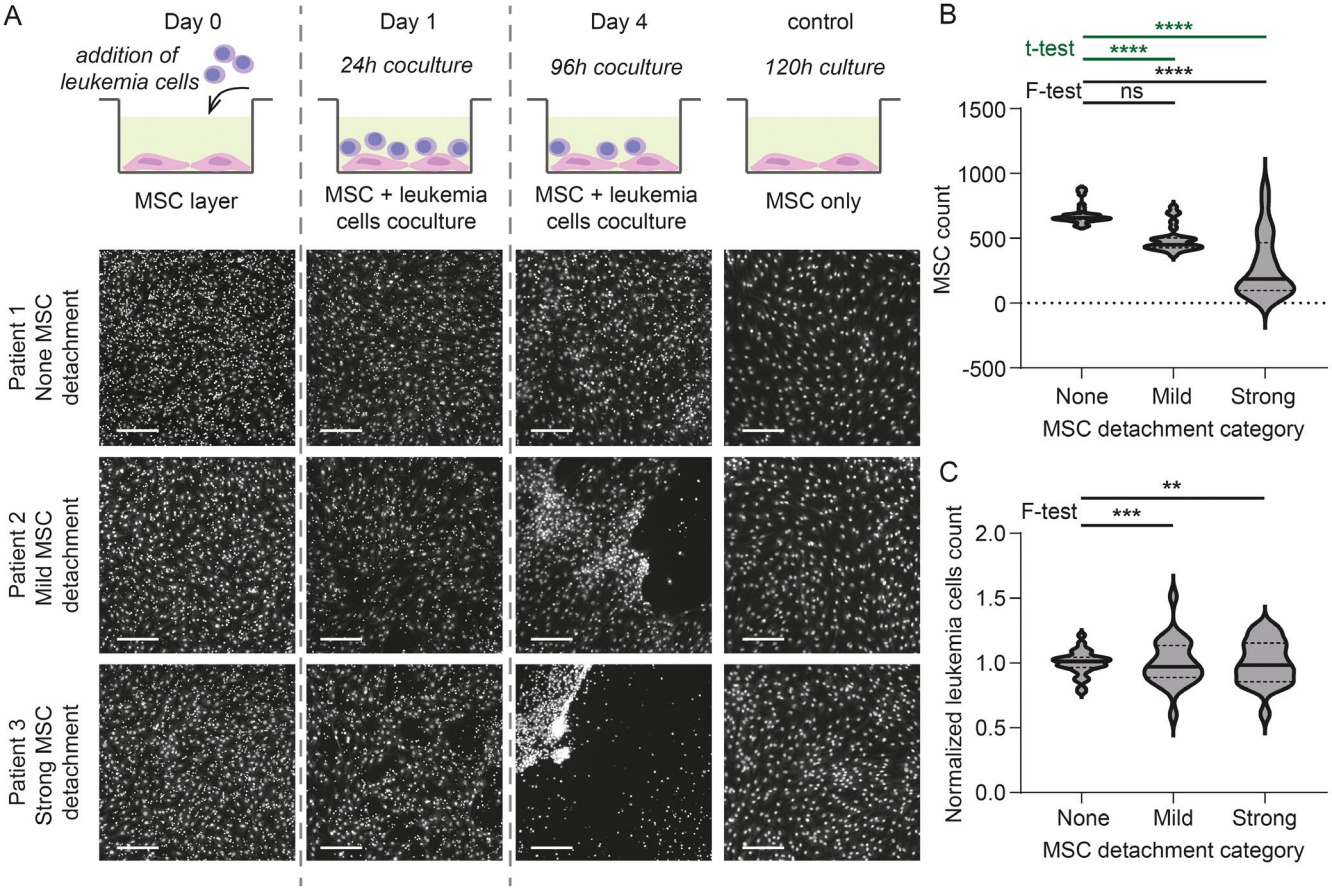
Coculture of MSC and leukemia cells can cause MSC detachment. (A) The scheme on top shows the course of the MSC‐leukemia cells coculture. Images below show CQD‐stained cell nuclei in each stage of the experiment using an example of three patient samples—causing no MSC detachment (top), mild MSC detachment (middle), or strong MSC detachment (bottom). Scale 200 μm. Leukemia types used: Patient 1, T‐ALL; Patient 2, T‐ALL; and Patient 3, ALAL. (B) MSC were counted from the MSC‐leukemia cell cocultures on day 4. For each category, MSC counts contain combined data from 2 to 4 patients (None *n* = 32, Mild *n* = 25, and Strong *n* = 24); asterisks indicate the statistical significance of the *t*‐test (in green) and *F*‐test to compare variances (in black): ****p < 0.0001; ns not significant. Leukemia types used in MSC detachment categories: None—B‐ALL, T‐ALL, AML, ALAL; Mild—B‐ALL, T‐ALL; Strong—AML, AML, ALAL. (C) Normalized leukemia cells were counted from the MSC‐leukemia cells coculture on day 4. For each category, leukemia cell counts contain combined data from 2 to 4 patients (None *n* = 32, Mild *n* = 25, and Strong *n* = 24); asterisks indicate the statistical significance of the *F*‐test to compare variances: ***p < 0.001; **p < 0.01. Means were not compared statistically. Leukemia types used in MSC detachment categories: None—B‐ALL, T‐ALL, AML, ALAL; Mild—B‐ALL, T‐ALL; Strong—AML, AML, ALAL. ALAL, acute leukemias of ambiguous lineage; AML, acute myeloid leukemia; B‐ALL, B lymphoblastic acute leukemia; CQD, CyQUANT Direct Cell Proliferation Assay; MSC, mesenchymal stromal cells; T‐ALL, T lymphoblastic acute leukemia.

Coculture of MSC with leukemia cells simulates the bone marrow environment, and data from cocultures reflected in vivo drug responses more closely than without stromal support [7]. However, sometimes, the addition of leukemia cells leads to MSC detachment from the plastic surface of the well bottom. To the best of our knowledge, this phenomenon has not yet been described in the literature. However, it is known in the DRP community to happen rather frequently. In our hands, it occurred in 27.8% of experiments (5 of 18). The detachment could be either mild, with a few larger holes in an otherwise compact MSC layer (11.1% of cases, 2 of 18), or strong, starting with holes in the MSC layer within the first 24 h of coculture and ending with the majority of MSC detached and forming dense patches consisting of a mixture of MSC and leukemia cells (16.7% of cases, 3 of 18; Figure 1A). Leukemia cells indeed appeared to be the trigger of the detachment because we never observed the detachment in control wells containing only MSC (among 18 plates, each containing a minimum of 18 control wells). The tendency to force MSC detachment is an inherent feature of a given specimen, and although most cases were observed with samples from AML patients, it is not limited to any leukemia type (Figure S2A). However, the MSC detachment was not always consistently reproduced when a different aliquot of the same individual was tested under the same experimental conditions (Figure S2B). In the absence of detachment, MSC form a monolayer at the bottom of each well, and automated image analysis, therefore, easily detects and distinguishes them from leukemia cells that lie on top of them. With an increasing degree of MSC detachment, the number of detected MSC drops and variance among replicates significantly increases (Figure 1B). The stronger the MSC detachment, the more variable are also the numbers of detected viable leukemia cells in replicates (Figure 1C). The source of these discrepancies lies primarily in image analysis because individual MSC are challenging to detect or accurately separate from leukemia cells in stretches and dense patches formed after MSC detachment (Figure S3). In addition, the negative impact of the MSC detachment on their role as support for leukemia cells can be expected.

### 3.2 Use of Conditioned Medium Prevents MSC Detachment

**FIGURE 2:**
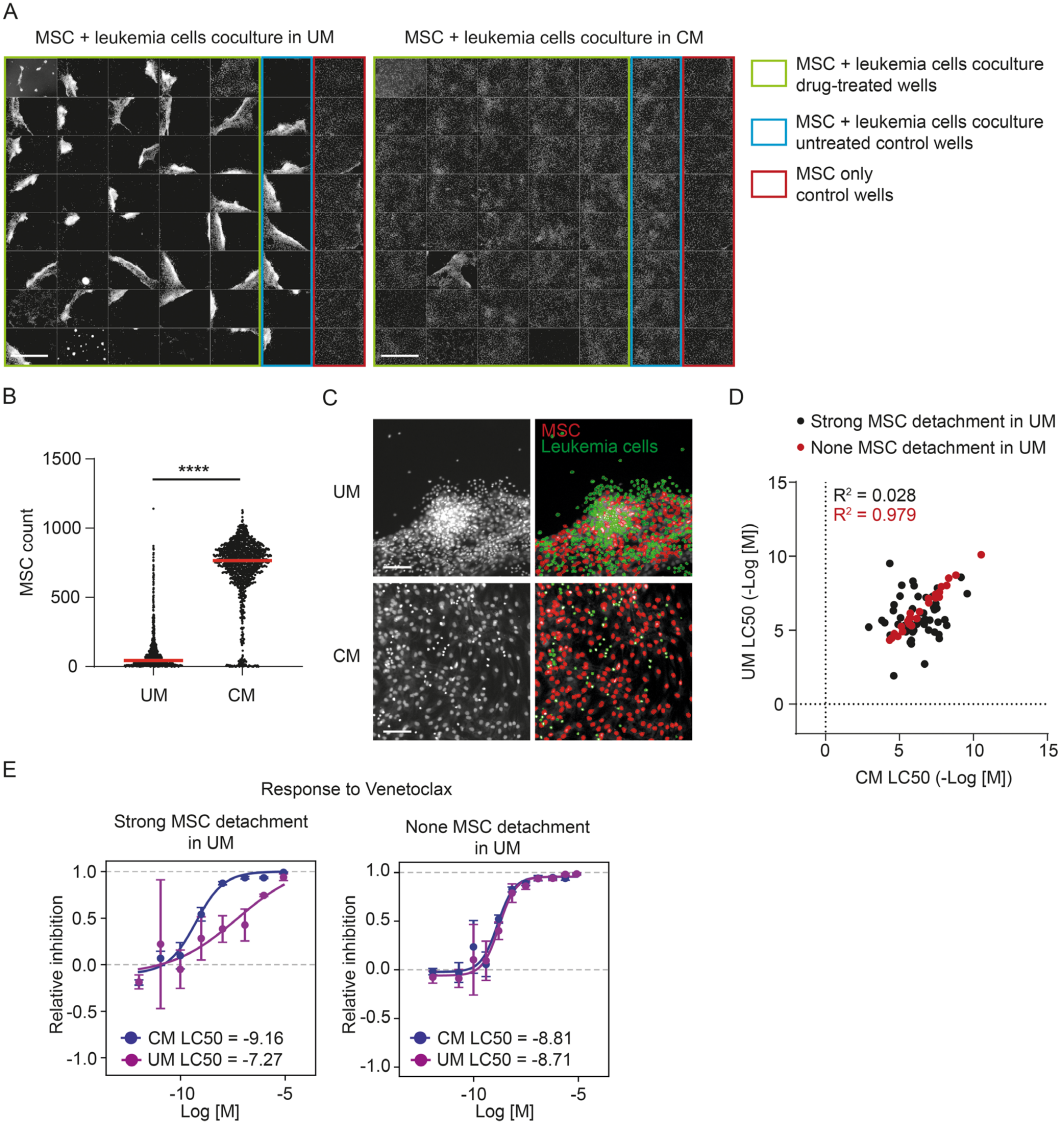
Addition of conditioned medium to MSC‐leukemia cells coculture prevents MSC detachment. (A) Selected fields from 1536‐well plates used for the parallel DRP experiment. CQD‐stained cell nuclei were imaged on day 4 of the experiment. The MSC‐leukemia cells coculture using the leukemia cells from patient 4 (leukemia type AML) was performed in unconditioned medium (UM, left) or conditioned medium (CM, right). Scale 1 mm. (B) Distribution of MSC counts from all wells in 1536‐well plates. *t*‐test ****p < 0.0001. (C) Examples of automated detection of live MSC (red) and leukemia cells (green) during automated image analysis of CQD‐stained cell nuclei from day 4 of coculture. Scale 100 μm. (D) Correlation of corresponding LC50 values calculated for all compounds tested on leukemia cells from patient 4 (leukemia type AML) in the DRP either in conditioned (CM) or unconditioned (UM) medium. Black dots represent data from an experiment with strong MSC detachment in unconditioned medium (R^2^ = 0.028), and red dots represent data from an experiment with no MSC detachment in unconditioned medium (R^2^ = 0.979). (E) A comparison of dose–response profiles for Venetoclax performed in conditioned (CM) or unconditioned (UM) medium. Each data point represents a mean +/− SD calculated from duplicates. The left plot represents data from an experiment with strong MSC detachment in unconditioned medium; the right plot represents data from an experiment with no MSC detachment in unconditioned medium. In both cases, leukemia cells from patient 4 (leukemia type AML) were used. AML, acute myeloid leukemia; CM, conditioned medium; CQD, CyQUANT Direct Cell Proliferation Assay; DRP, drug response profiling; MSC, mesenchymal stromal cells; UM, unconditioned medium.

To overcome the problem with MSC detachment, we introduced the utilization of MSC‐conditioned medium in the DRP. We observed that replacing one third volume of fresh screening medium with screening medium conditioned on MSC for 3 days positively affected MSC attachment to the well bottom in the coculture, and MSC then remained in monolayer. Parallel DRP experiments using the same patient sample in either unconditioned or conditioned medium showed a markedly reduced MSC detachment in conditioned medium (Figure 2A,B). The evenly distributed monolayer of MSC in conditioned medium facilitated automated image analysis. Thus, more precise numbers of viable leukemia cells could be extracted in comparison with unconditioned medium, where strong MSC detachment occurred in the majority of wells (Figure 2C). This translated into more reliable response curves describing the drug's effect on leukemia cell viability. In general, the correlation of corresponding drug responses measured in both conditioned medium and unconditioned medium was poorer in case strong MSC detachment occurred in unconditioned medium compared to the situation when MSC stayed attached (Figure 2D). A detailed view of differences in dose–response curves using an example of Venetoclax is shown in Figure 2E. This clearly illustrates the negative effect that MSC detachment has on the precision of DRP assay results.

**FIGURE 3:**
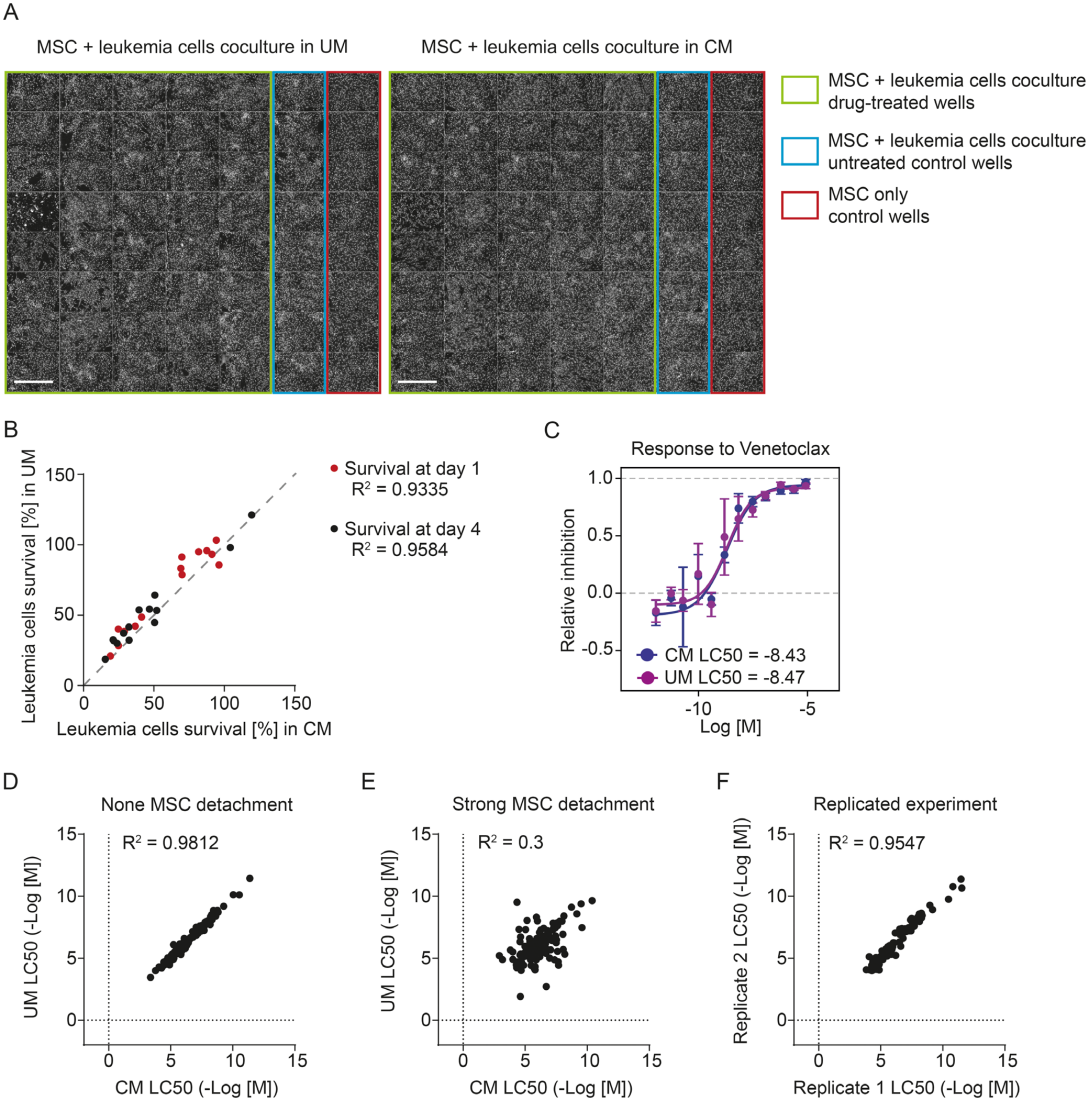
Use of conditioned medium does not affect the DRP output perse. (A) Selected fields from 1536‐well plates used for the DRP experiment. CQD‐stained cell nuclei were imaged on day 4 of the experiment. The MSC–leukemia cells coculture using the leukemia cells from patient 5 (leukemia type B‐ALL) was performed in unconditioned medium (UM, left) or conditioned medium (CM, right). Scale 1 mm. (B) Correlation of proportion of surviving leukemia cells after 24 h (red) or 96 h (black) of coculture in conditioned (CM) or unconditioned (UM) medium (data from 14 parallel experiments of nine patients; leukemia types: 1× AML, 5× B‐ALL, and 3× ALAL). (C) A comparison of dose–response profiles for Venetoclax performed in conditioned (CM) or unconditioned (UM) medium, with no MSC detachment in UM (data from the experiment shown in panel A). (D) Correlation of corresponding LC50 values calculated for all compounds tested on leukemia cells in the DRP in conditioned (CM) or unconditioned (UM) medium. Data are pooled from eight DRPs of four patients (*n* = 152) with no MSC detachment in all plates with UM. Leukemia types used: 1 × ALAL, 1 × AML, and 2× B‐ALL. (E) Correlation of corresponding LC50 values calculated for all compounds tested on leukemia cells in the DRP in conditioned (CM) or unconditioned (UM) medium. Data are pooled from six DRPs of two patients (*n* = 143) with strong MSC detachment in all plates with UM. Leukemia types used: 2× AML. (F) Correlation of corresponding LC50 values calculated for all compounds tested in two independent DRPs of the same patient performed in conditioned medium. Data are pooled from eight DRPs of four patients (*n* = 106). Leukemia types used: 2× B‐ALL, 2× ALAL. ALAL, acute leukemias of ambiguous lineage; AML, acute myeloid leukemia; B‐ALL, B lymphoblastic acute leukemia; CM, conditioned medium; CQD, CyQUANT Direct Cell Proliferation Assay; DRP, drug response profiling; MSC, mesenchymal stromal cells; UM, unconditioned medium.

To ensure that conditioned medium does not alter leukemia cell viability per se and that the drug responses were not directly affected, we performed parallel experiments using leukemia cells that did not cause any MSC detachment (Figure 3A). The addition of conditioned medium resulted in a mild change in pH of the medium at the start of the coculture (conditioned medium had pH 7.65, unconditioned medium had pH 7.81), but the overall leukemia cell viability was not affected by the use of conditioned medium. On day 1, cells in unconditioned medium showed slightly higher viability than those in conditioned medium, but on day 4, cells showed a similar survival in both media (Figure 3B). A possible explanation is that conditioned medium contains fewer nutrients and thus provides weaker support for freshly thawed cells, but in later stages of the assay, it supports cells equally well as unconditioned medium. The corresponding response curves calculated from DRPs measured in both media without any MSC detachment were in much better agreement compared to those calculated from experiments where strong MSC detachment occurred in unconditioned medium (Figure 3C–E). In addition, the correlation in this case was very close to the correlation of results from independently repeated DRPs (Figure 3F). Therefore, we can conclude that implementing conditioned medium has no adverse effect on the leukemia cells' viability or their responses to drugs.

## 4 Discussion

**FIGURE 4:**
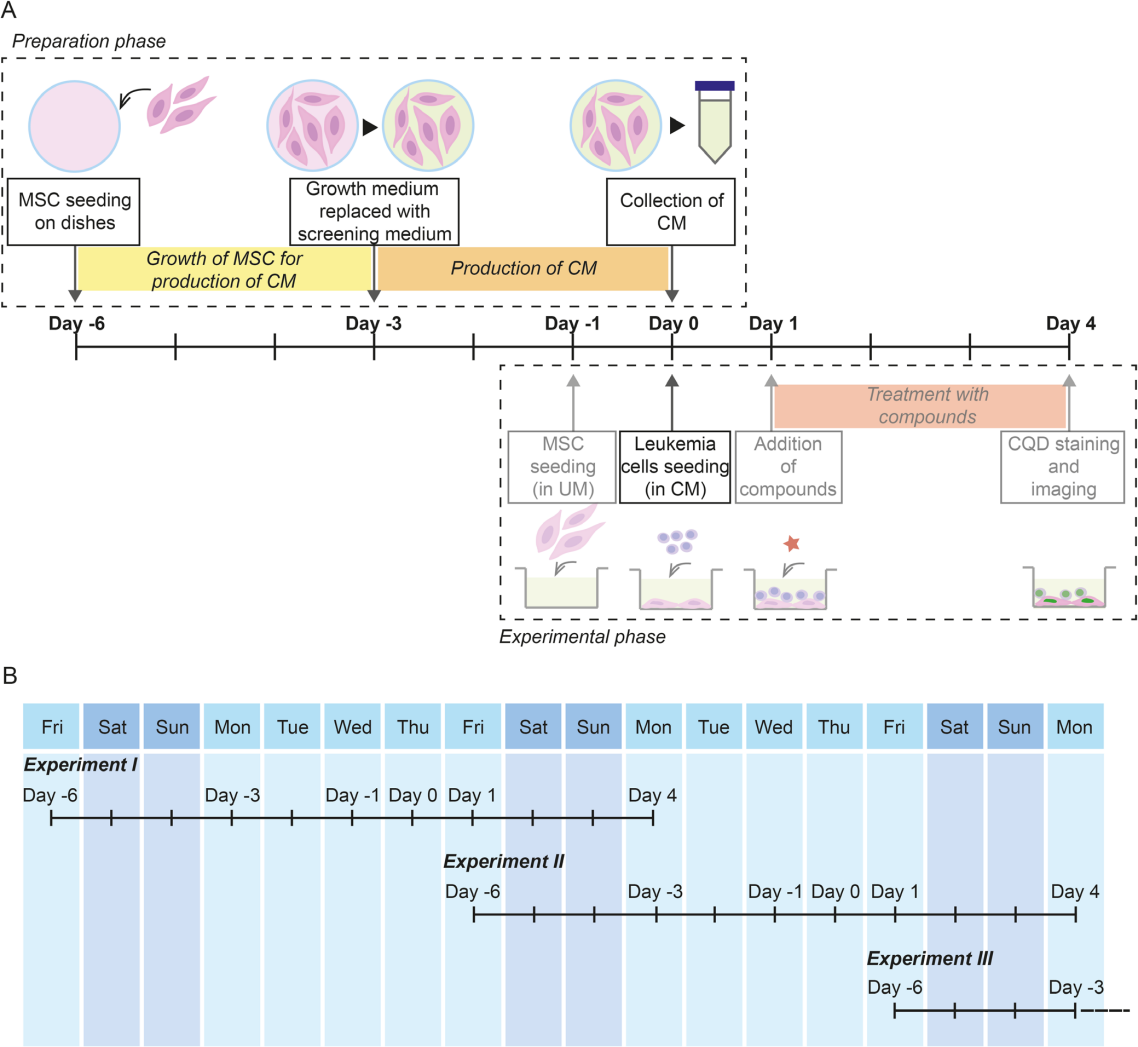
Upgraded pipeline of DRP experiment. (A) A scheme of the experimental DRP pipeline, with all critical steps indicated on a timeline showing the coupling of conditioned medium production with the experimental part. The preparation phase (the top part of the scheme) covers the production of MSC‐conditioned medium. The experimental phase (the bottom part of the scheme) covers the DRP procedure itself. The experimental phase is identical to the DRP procedure depicted in Figure S1 with one exception, which is the addition of leukemia cells in conditioned medium in this upgraded pipeline. (B) Functional scenario on how to link up individual experiments for continuous DRP screening using the upgraded pipeline. CM, conditioned medium; CQD, CyQUANT Direct Cell Proliferation Assay; DRP, drug response profiling; MSC, mesenchymal stromal cells; UM, unconditioned medium.

In this study, we demonstrated that unwanted MSC detachment caused by the addition of leukemia cells into the coculture can be prevented by the simple addition of MSC‐conditioned medium to the coculture. Before introducing conditioned medium to the DRP pipeline, we observed strong MSC detachment in 16.7% of DRPs (3 cases out of 18). Upgrading the pipeline using conditioned medium reduced cases of strong detachment to 3.3% of DRPs (2 cases out of 60; one AML, one ALAL). Unfortunately, we failed to identify the source of the persisting MSC detachment in these two cases. Nevertheless, despite there being still some cases of MSC detachment that cannot be rescued by conditioned medium, standard use of conditioned medium in DRP assay would, in general, contribute to the collection of more precise data, which is critical for predicting in vivo responses of individual patients. Implementing the use of conditioned medium in a standard DRP pipeline is illustrated in Figure 4A. It means no problems even for large continuous screening campaigns because it can be setup with overlapping preparation and experimental phases to save time (Figure 4B). It is possible that specific surface coating may improve MSC surface attachment [6], and therefore also increase the resistance of MSC monolayer to the leukemia cell‐triggered detachment. However, surface pre‐coating would unnecessarily complicate the established DRP protocol, let alone being largely impossible to apply in small‐scale formats (i.e., 1536‐well plate). Compared to this, our solution using conditioned medium means only a minor change in the established protocol and a minimal increase in total costs.

Our experience so far suggests that despite any leukemia type being capable of triggering MSC detachment, it is more likely to occur in a coculture with AML cells. However, our current collection of experiments with MSC detachment is not large enough to draw statistically significant conclusions. Further investigation is needed to confirm if there are indeed any leukemia types more prone to causing MSC detachment. Molecular causes of MSC detachment and the mechanism of its rescue by conditioned medium have yet to be elucidated. Multiple biologically active molecules have been shown to be secreted by MSC. Among the most abundant components of the MSC secretome were identified various extracellular matrix proteins (e.g., collagens, fibrillins) [8]. Increased levels of extracellular matrix proteins in conditioned medium could contribute to better adherence of MSC to the surface and their increased resistance to leukemia‐cell‐driven MSC detachment. MSC also secrete a number of cytokines, chemokines, and growth factors (e.g., VEGFC, TGF‐β1, TGF‐ β2, GDF6, SDF1, and IL‐6) that can further modulate the interaction between leukemia cells and MSC [8, 9]. In addition, MSC are known to produce extracellular vesicles—nano‐ to micro‐sized lipid bilayer membrane particles containing various functional proteins (e.g., cytokines, cytoskeletal proteins, and heat‐shock proteins) but also nucleic acids (e.g., miRNA and mRNA) and lipids—that have been shown to be involved in tissue regeneration and are considered a promising cell‐free therapeutic tool [10, 11, 12]. Based on the conditioned medium preparation protocol, we assume it contains extracellular vesicles and thus could modulate MSC‐leukemia cell interaction also through them. However, although it is plausible that the rescue effect we observe originates from MSC‐secreted molecules or extracellular vesicles, evidence for this mechanism needs to be proven.

## 5 Conclusions

In conclusion, by upgrading the protocol for ex vivo DRP of leukemia cells with the introduction of MSC‐derived conditioned medium, we provide a simple solution to frequently observed MSC feeder layer detachment and, therefore, secure accurate results for the vast majority of patient samples. Despite so far missing knowledge about the precise mechanism of MSC detachment rescue by conditioned medium, patients with leukemia may already benefit from the improved precision of the DRP results and thus a more accurate prediction of their in vivo response to drugs. Thanks to its simplicity, the upgrade is also easily implemented into high‐throughput DRP screens that are vital for leukemia cell research in general.

## Author Contributions

**Ivana Vonkova:** conceptualization (equal), methodology (lead), investigation (equal), formal analysis (equal), data curation (equal), project administration (lead), funding acquisition (equal), writing – original draft (equal), writing – review and editing (equal) and visualization (lead). **Olga Martinkova:** investigation (equal) and writing – review and editing (equal). **Jitka Stancikova:** conceptualization (equal), resources (equal), writing – original draft (equal), and writing – review and editing (equal). **Ctibor Skuta:** formal analysis (equal), data curation (equal), and writing – review and editing (equal). **Ondrej Hrusak:** conceptualization (equal), resources (equal), funding acquisition (equal), supervision (equal), and writing – review and editing (equal). **Petr Bartunek:** conceptualization (equal), funding acquisition (equal), supervision (equal), and writing – review and editing (equal).

## Ethics Statement

The study was approved by the Ethical Committee of the Second Faculty of Medicine, Charles University in Prague (ref. no. EK‐602.32/22; agreement emitted on May 15, 2022) and conducted in accordance with the Declaration of Helsinki. Informed consent was obtained from all individuals included in this study, or their legal guardians or wards.

## Conflicts of Interest

The authors declare no conflicts of interest.

## Supporting information


**Figure S1.** General pipeline of DRP experiment.

**Figure S2.** Examples and reproducibility of leukemia cells derived MSC detachment.

**Figure S3.** Automated detection of MSC and leukemia cells in cases of various levels of MSC detachment.

**Table S1.** Overview of samples used in experiments presented in the study.

## Data Availability

The data that support the findings of this study are available from the corresponding author upon reasonable request.
