# Supplementary material for: Feeder Cell Detachment in Drug Response Profiling of Leukemia Cell Coculture Can Be Prevented by Conditioned Medium
Source: Cancer Med. 2025 Jul 19;14(14):e71070. doi: 10.1002/cam4.71070 (PMC12274628; doi:10.1002/cam4.71070)
Supplement: Supplementary file 1 — Figure S1. General pipeline of DRP experiment. [file CAM4-14-e71070-s003.pdf]

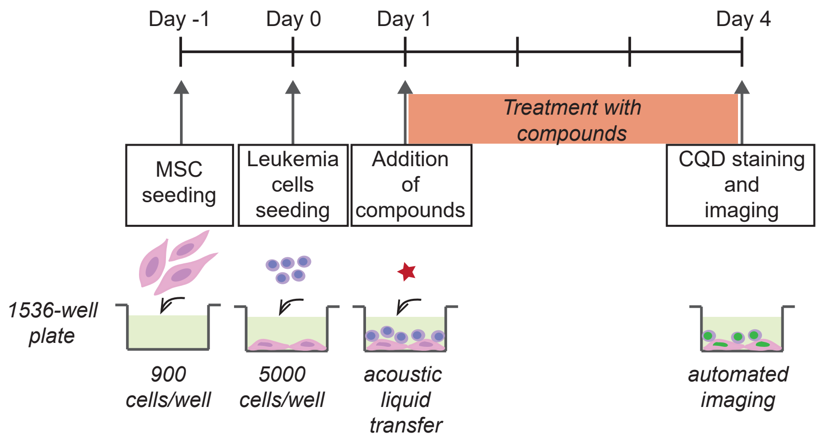

Figure S1 General pipeline of DRP experiment
A scheme of an experimental pipeline of DRP with all key steps indicated on a timeline. MSC, mesenchymal stromal cells; DRP, drug response profiling; CQD, CyQUANT Direct Cell Proliferation Assay
