# Supplementary material for: Feeder Cell Detachment in Drug Response Profiling of Leukemia Cell Coculture Can Be Prevented by Conditioned Medium
Source: Cancer Med. 2025 Jul 19;14(14):e71070. doi: 10.1002/cam4.71070 (PMC12274628; doi:10.1002/cam4.71070)
Supplement: Supplementary file 2 — Figure S2. Examples and reproducibility of leukemia cells derived MSC detachment. [file CAM4-14-e71070-s002.pdf]

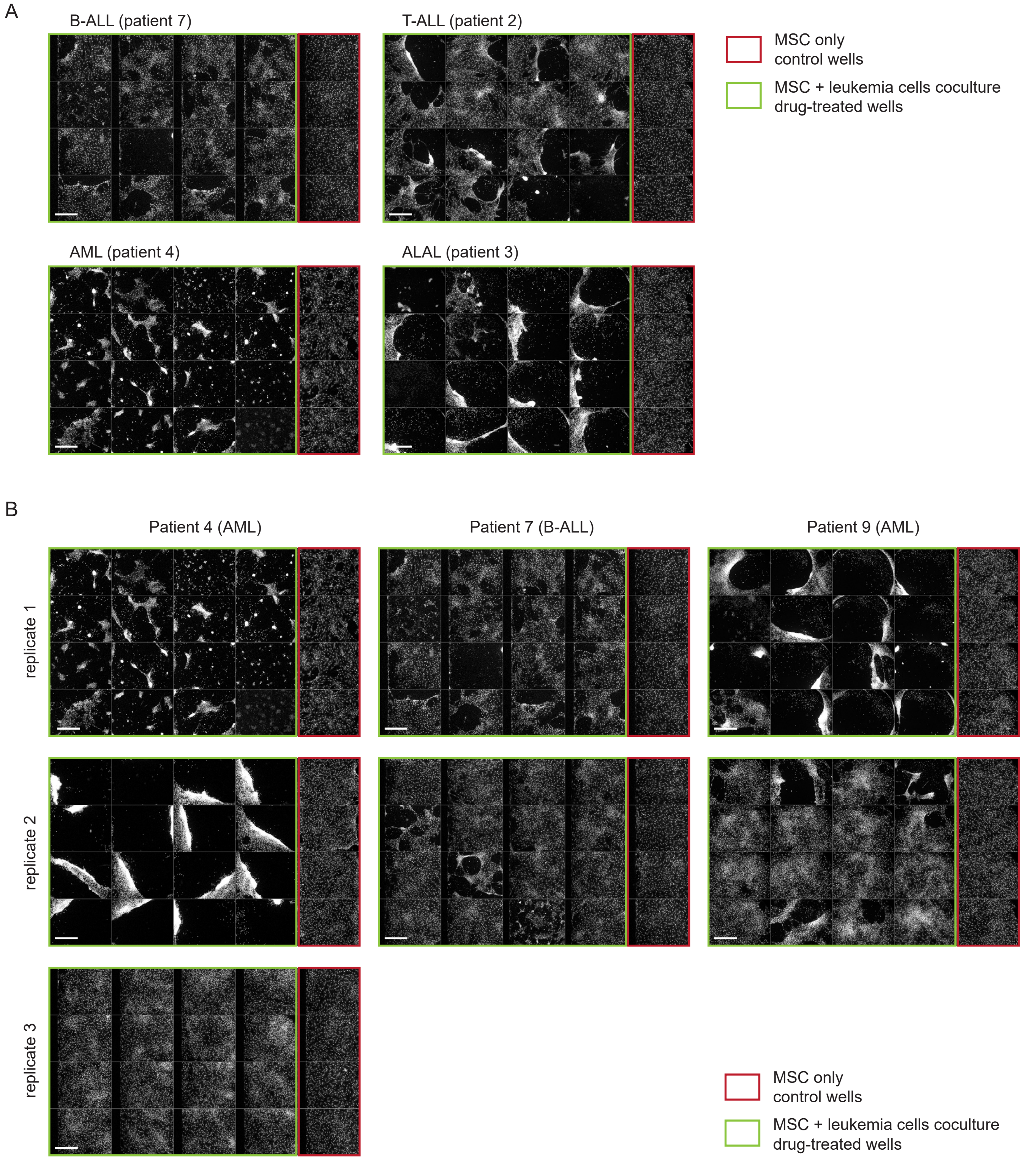

Figure S2 Examples and reproducibility of leukemia cells derived MSC detachment
Image fields from 1536-well plates used for the DRP experiment. CQD-stained cell nuclei were imaged on day 4 of the experiment. Each patient is identified by number and by leukemia type. Scale 500 μm.
(A) Examples of mild (patient 7, B-ALL) and strong (patient 2, T-ALL; patient 3, ALAL; patient 4, AML) MSC detachment.
(B) Reproducibility of MSC detachment. The MSC-leukemia cells coculture using leukemia cells from patient 4 (leukemia type AML, left column), patient 7 (leukemia type B-ALL, middle column) or patient 9 (leukemia type AML, right column) performed in unconditioned medium. Results of two or three independent experiments are shown (replicate 1, 2 and 3).
MSC, mesenchymal stromal cells; DRP, drug response profiling; CQD, CyQUANT Direct Cell Proliferation Assay; T-ALL, T lymphoblastic acute leukemia; B-ALL, B lymphoblastic acute leukemia; AML, acute myeloid leukemia; ALAL, acute leukemias of ambiguous lineage
