# Supplementary material for: Feeder Cell Detachment in Drug Response Profiling of Leukemia Cell Coculture Can Be Prevented by Conditioned Medium
Source: Cancer Med. 2025 Jul 19;14(14):e71070. doi: 10.1002/cam4.71070 (PMC12274628; doi:10.1002/cam4.71070)
Supplement: Supplementary file 3 — Figure S3. Automated detection of MSC and leukemia cells in cases of various levels of MSC detachment. [file CAM4-14-e71070-s004.pdf]

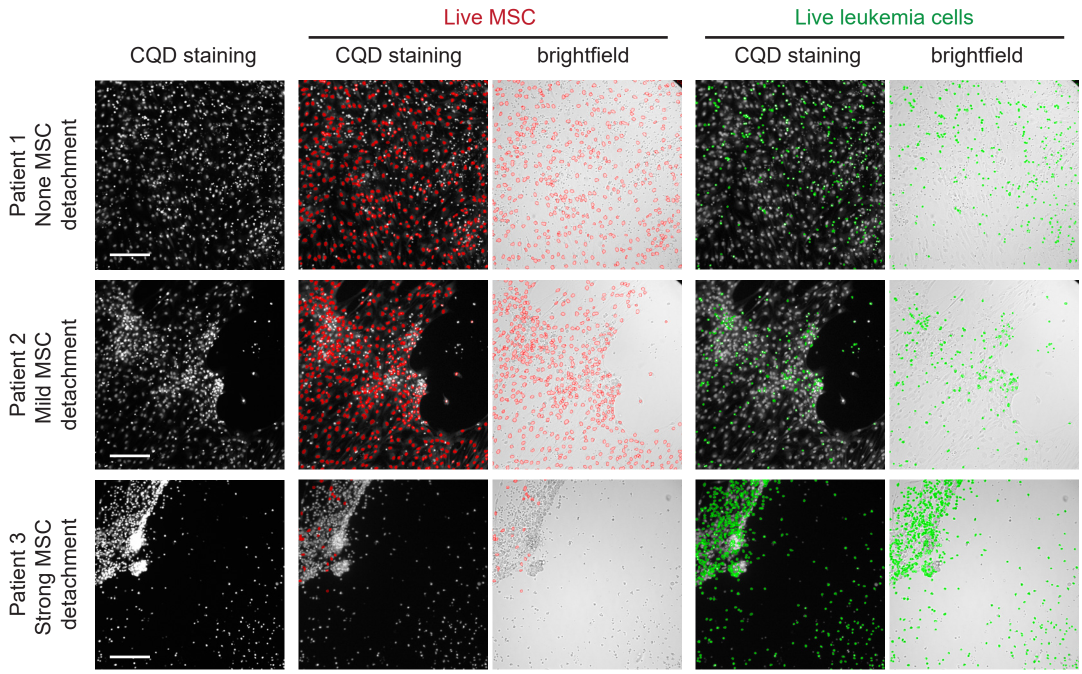

Figure S3 Automated detection of MSC and leukemia cells in cases of various levels of MSC detachment
CQD-stained (nuclei staining) MSC-leukemia cell cocultures at day 4, as shown in Figure 1. The left column shows raw images from all three cases (none, mild, and strong detachment). Two middle columns show automatically detected MSC (red) in the fluorescent image (left) and in the brightfield image (right). Two columns on the right side show automatically detected leukemia cells (green) in fluorescent image (left) and in brightfield image (right). Scale 200 μm. Leukemia types used: patient 1, T-ALL; patient 2, T-ALL; patient 3, ALAL.
MSC, mesenchymal stromal cells; CQD, CyQUANT Direct Cell Proliferation Assay; T-ALL, T lymphoblastic acute leukemia; ALAL, acute leukemias of ambiguous lineage
